# Mycological Investigation of Bottled Water Dispensers in Healthcare Facilities

**DOI:** 10.3390/pathogens10070871

**Published:** 2021-07-10

**Authors:** Zsófia Tischner, Rózsa Sebők, László Kredics, Henrietta Allaga, Márta Vargha, Ágnes Sebestyén, Csaba Dobolyi, Balázs Kriszt, Donát Magyar

**Affiliations:** 1 Department of Environmental Safety, Institute of Aquaculture and Environmental Safety, Hungarian University of Agriculture and Life Sciences, H-2100 Gödöllő, Hungary; dobolyi.csaba@mkk.szie.hu (C.D.); kriszt.balazs@szie.hu (B.K.); 2 National Public Health Center, H-1097 Budapest, Hungary; vargha.marta@nnk.gov.hu (M.V.); sebestyen.agnes@nnk.gov.hu (Á.S.); magyar.donat@gmail.com (D.M.); 3 Department of Microbiology, Faculty of Science and Informatics, University of Szeged, H-6726 Szeged, Hungary; kredics@bio.u-szeged.hu (L.K.); henrietta.allaga@gmail.com (H.A.)

**Keywords:** water quality, water cooler, microorganisms, molds, bacteria, health risks

## Abstract

The usage of bottled water dispensers (BWDs) has spread worldwide. Despite their popularity, few studies have dealt with their microbial contaminants, and little attention is given to their fungal contamination. To our knowledge this is the first mycological study of BWDs in Europe. 36 devices have been examined in Budapest, Hungary. Despite of the strictly regulated water hygiene system in Hungary, molds and yeasts were detected in 86.8% of the samples, 56.76% were highly contaminated. Elevated heterotrophic plate counts were also observed in all samples compared to that of Hungarian drinking water. As all physical and chemical water quality characteristics have met the relevant national and European parametric values and neither totally explained the results of microbial counts, the effect of usage and maintenance habits of the devices were examined. Fungal concentrations were affected by the time elapsed since disinfection, days remaining until expiration of bottles, month of sampling and exposure to sunlight during storage. Microbes are able to proliferate in the bottled water and disperse inside the BWDs. Many of the detected fungal species (*Sarocladium* *kiliense*, *Acremonium* *sclerotigenum*/*egyptiacum*, *Exophiala* *jeanselmei* var. *lecanii*-*corni*, *Exophiala* *equina*, *Meyerozyma* *guilliermondii*, *Cystobasidium* *slooffiae*, *Aspergillus* *jensenii*, *Bisifusarium* *biseptatum*) are opportunistic pathogens for subpopulations of sensitive age groups and patients with immunodeficient conditions, including cystic fibrosis. Thus BWDs may pose a health risk to visitors of healthcare institutions, especially to patients with oral lesions in dental surgeries. The study draws attention to the need to investigate microbial contamination of these devices in other countries as well.

## 1. Introduction

Water is a necessary basis of life on earth and the provision of high-quality drinking water is essential. The WHO Drinking Water Quality Guidelines [1] recommend to consider microbiological, disinfection, chemical and radiological aspects, as well as organoleptic characteristics that affect the acceptance of water by consumers. The organization has collected and characterized the major factors posing microbiological risk, but did not set any guideline values, as the hazard, presented by the individual pathogens, varies by geographical area and population. Parametric values only apply to fecal indicators (0/100 mL). WHO also discusses opportunistic pathogenic organisms that may be present in the environment, but are only able to cause disease in certain vulnerable subpopulations (i.e., elderly, minors, pregnant women, people with burns and extensive injuries and immunocompromised patients). A list of pathogenic microorganisms which require monitoring is provided by the WHO Guidelines [1]. The European Union Drinking Water Directive sets parametric value for further bacteria, but not for fungi [2]. Direct detection of fungi by culture is required solely by Swedish legislation in Europe (limit value to 100 CFU/100 mL) [3]. Some countries (such as Hungary and the Czech Republic) require microscopic investigation of drinking water and have set separate parametric values for groups of organisms: in Hungary, for fungi 0 individuals/L parametric value applies [4].

Relatively large amounts of biodegradable organic matter [1], warm or ambient temperature water [5] and low concentration of residual chlorine may lead to the proliferation of these organisms [1]. Bottled water might contain tap water, spring water or mineral water and consequently it is covered by various regulations in different countries, such as the drinking water legislation or food regulation. Most international bodies recommend using risk-based approaches for the production of bottled water, such as water safety planning or HACCP [6,7,8].

**Figure 1:**
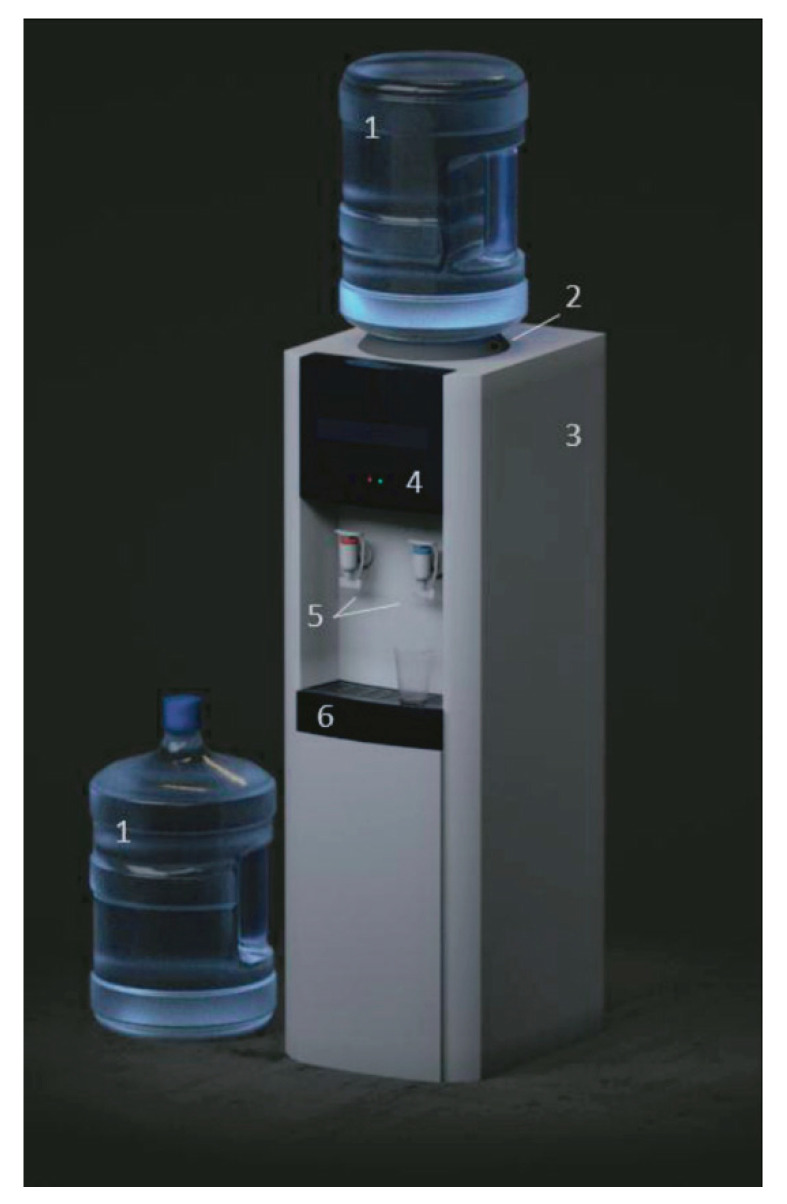
Assembly of bottled water dispensers. 1: bottle, 2: air filter, 3: hot and cold water tanks (inside of the device), 4: pipeline (inside of the device), 5: hot and cold taps, 6: drip tray. (3D graphics is courteously from Pavel Zosim).

Fungal contamination of water contained in modern household devices, such as washing machines [9] and dishwashers [10,11] have attracted attention in the last decade, as they provide new habitats for microscopic fungi. Bottled water dispensers (BWDs, also known as water coolers, Figure 1) were rarely studied, the only study on them was carried out by Yamaguchi et al. [12] in residents and office buildings in Brazil. These devices are intensively used in public buildings, workplaces and in health institutions worldwide. However, in several studies fungal contaminations of bottled water were detected [3,12,13,14,15,16].

In BWDs bottled water is supplied by a system of pipes, filters and taps. The simplest version of BWDs just cools the water, but often it is also able to heat it with a separate pipe system. Some devices provide carbonated water as well.

Previous studies demonstrated fungal contamination of drinking water and consequent health problems [3,17,18,19,20]. A recent study draws attention to the problem of pathogenic fungal contamination of tap water in hospitals [21]. Their results raise the question whether or not fungi can contaminate BWDs as well with a risk mostly to people using them in hospitals. The aim of the current research was to examine the mycological composition of BWDs operating in healthcare facilities (hospitals, pharmacies and dental clinics) in Budapest, Hungary.

## 2. Results

### 2.1. Fungal Statistics

The 56.76% of sampled water from BWDs was highly contaminated with fungi (>6 CFU/mL). High concentration of filamentous fungi and yeasts was detected in 51.35% and 29.73% of the water from the devices, respectively. The most important relationships of the total fungal counts are the pH, time since the last maintenance or disinfection and the temperature of water. Significance, correlation coefficients and components of total fungal values responsible for the relationship are listed in Table 1.

Filamentous fungal counts obviously correlated with the number of yeasts in water samples, furthermore, with the filamentous fungal results of swab samples collected from the taps of BWDs. Expiration of the bottles (Figure S1), last maintenance or disinfection, TOC, month of sampling (Figure S2), exposure to sun and additional parameters, as listed in Table S1 also have been significantly related.

Number of yeasts correlated with nitrite ion concentration and the ordered quantity of the bottles was also a significant parameter, as shown in Table S2 containing the significant relationships of yeast counts of water samples.

The significant correlations of swab samples collected from drip trays are included in Table S3. Volume flow (Figure S3), detectable nitrite ion concentration of water, the exposure of bottles to sun during storage and presence of pseudo hardness in water had a significant effect on filamentous fungi. Yeast counts were affected by the site of operation of the device (i.e., on corridor or not) and by the extra disinfection by personnel besides the obligatory one during maintenance.

The total fungal counts of swab samples from taps significantly differed between the devices of two manufacturers (of the total 9 manufacturers) (Table S4). Alkalinity had the main effect on yeast counts.

In the water and swab samples a total of 81 fungal isolates were identified, respectively. Fungal taxa found in the samples are listed in Table 2.

### 2.2. Physical, Chemical and Biological Descriptional Statistics

According to the questionnaire-based survey, no complaints have been reported about the organoleptic characteristics of the water provided by the BWDs. Organoleptic testing during sampling confirmed this finding. In total 58% of the sampled water bottles contained mineral water which was not treated or disinfected.

Table S5 contains the descriptive statistics of the measured numerical variables. In most cases the concentration of the nitrogen forms was under the limit of detection, nitrite ion was detected in 8.1% and ammonium ion in 13.5% of the water samples. Pseudo hardness (carbonate hardness belonging to monovalent cations) was present in 24.3% of the water samples.

The HPC results were high, in several cases (19/37 at 22 °C and 14/37 at 37 °C) exceeding the upper limit of quantification (10,000 CFU/mL). *Pseudomonas aeruginosa* was detected in 10.8% of the dispensers.

The HPC values incubated at 22 and 37 °C correlated (*p* = 8 × 10^−11^; R = 0.84). Both of them had positive relationships with the TOC results (*p* = 0.0023; R = 0.5 at 22 °C and *p* = 0.0014; R = 0.52 at 37 °C, Figure S4) and a negative correlation with the number of days remaining until the expiration date of the bottles (*p* = 0.0295; R = −0.37 at 22 °C and *p* = 0.0458; R = −0.34 at 37 °C, Figure S1) according to Pearson correlation. The HPC at 37 °C also correlated with the number of days since the last maintenance and/or disinfection (*p* = 0.0478; R = −0.43). The log transformed total HPC results correlated negatively with the filamentous fungal counts of the water samples (*p* = 0.036, R = −0.38) (Figure S5).

The month of sampling (*p* = 0.0051 at 22 °C and *p* = 0.0004 at 37 °C, Figure S2), the type of the institution (*p* = 0.002 at 22 °C and *p* = 0.0012 at 37 °C, Figure S6) and the frequency of the disinfection process (*p* = 0.0159 at 22 °C and *p* = 0.0067 at 37 °C) affected the HPC values most significantly. Only 16.2% of the examined BWDs were disinfected more often than monthly, 18.9% every 1–2 month, 27% every 3–4 month, 2.7% every 5–6 month, 8.1% yearly and 24.3% less often than yearly. One of the examined BWDs was never disinfected.

BWDs used by the personnel had worse (*p* = 0.0252) and by customers had better (*p* = 0.0123) HPC results at 22 °C. Presumably it is connected to types of institutions, as BWDs used by customers with the lowest HPC counts operated mostly in pharmacies, while personnel used BWDs with the highest HPC counts mainly operated in private hospitals and dental clinics. BWDs containing air filter also were contaminated (*p* = 0.0136). If disinfection is performed by the staff besides the routine maintenance, both HPC values were better (*p* = 0.0011 at 22 °C, *p* = 0.0031 at 37 °C). BWDs installed in waiting areas had higher HPC values at 37 °C (*p* = 0.0491). Devices not exposed to any sunlight had better results at both temperatures (*p* = 0.0221 at 22 °C and *p* = 0.0336 at 37 °C). Furthermore, if the bottles had not been exposed to any sunlight during storage, it had a favorable effect on the HPC at 37 °C (*p* = 0.0407).

*P. aeruginosa* numbers [CFU/100 mL] correlated with the detected nitrite ion concentrations [mg/L] (*p* = 0.0016; R = 0.5) and with the number of yeasts in swab samples collected from the tap of BWDs (*p* = 0.0348; R = 0.35).

### 2.3. Questionnaire

Most of the sampled BWDs were located in hospitals: 37.8% in private and 18.9% in state hospitals, but some operated in dental clinics (27%) and pharmacies (16.2%). Samples were collected in April (35.1%), June (27%) and July (37.8%) of 2018. Most BWDs were positioned in the waiting rooms, on the corridors or in the kitchen (62.2%, 27% and 18.9%, respectively). Usually, devices are used by both the staff and patients (83.8% and 81.1%, respectively, or customers 16.2%). Some BWDs were able to provide carbonated water (24.3%), though we did not use this function during sampling. The examined BWDs were manufactured by eight different manufacturers (although 37.8% was the same brand), and 48.6% contained air filter according to their user’s manual. Usually, the disinfection process is part of the maintenance provided by the manufacturer or distributor, but in 18.9% the operating personnel also performs disinfection. Most of the BWDs were not exposed to sun (46.8%), 35.5% were affected by diffuse and 16.2% by direct sunlight, although only through a window. Bottles during storage were not exposed to sunlight in 64.9%, only to diffuse sunlight in 29.7% and to direct sunlight in 5.5% of the cases, also through the window glass. The quantity of bottles ordered at once was one to five (37.8%), six to ten (24.3%), eleven to twenty (21.6%) or even more bottles (16.2%), although some facilities where the sampling was performed operate more BWDs beside the one we collected information on. More than 94% of the sampled BWDs provided mineral water. Numerical variables are described in Table S6.

## 3. Discussion

BWDs are mainly made of plastic (PVC, PE, PB, PP), but also contain metal parts and rubber seals. Most of these are in contact with water, therefore bacteria and fungi are able to form biofilms on their surface and colonize these materials. The presence of various materials in a water system is known to enhance microbial growth and biofilm formation [35]. In this study, the authors did not investigate the effects on microbes caused by the different materials found in a BWD, as the biofilm forming properties had already been investigated by Pinto et al. [35]. We hypothesize that the accessibility of BWD parts (e.g., parts that are visible vs. parts that always remain hidden) could play a more important role in biofilm formation, than the BWD’s material itself.

The main objective of the study of Yamaguchi et al. [12] was the most similar to ours. However, their observations occurred in residential and office buildings in Brazil, while our study was carried out in healthcare institutions in a European country. There are higher risks of fungal infections via BWDs in hospitals and pharmacies where immunocompromised people may frequently use these devices or, multi-resistant yeasts can colonize surfaces of water-connected equipment. In the case of Yamaguchi et al. [12] it is not clear whether sampling water of BWDs occurred by opening the tap (water goes through the pipes of the device) or directly from the bottles. They found that the bottled mineral water was more contaminated than municipal tap water. The recovery of yeasts was significantly higher in bottled water than in tap water. Their observations were based on 20 L bottled mineral and tap water samples (*n* = 60), including fungal and bacterial (HPC, total coliform) investigations. The sampled water bottles in their study contained mineral water produced by a specific company. Such water cannot be treated nor added any exogenous elements. In our study we sampled 9 companies’ products. Five of them produced mineral water which was not disinfected. The others provided treated tap water or spring water. In our case, in total 58% of the sampled water bottles contained mineral water. They found *Candida* spp. as the most prevalent yeasts in the bottled water, including *C. parapsilosis* and *C. glabrata* as well. The authors highlighted that these species may cause nosocomial infections and raise the risk of candidaemia. In the last decade, multi-resistant *C. auris* (called also as ‘super yeast’) is an emerging problem in hospitals worldwide (including Europe) [36,37]. Such pathogen was detected in biofilms of catheters [38,39]. One can speculate that this fungus may colonize water pipes of BWDs as well. However, *Candida* species were relatively rare in our samples. The difference between Brazilian and Hungarian results remained unclear. Climate, as a factor may explain the difference between the results of these countries. Contrariwise to BWDs, pathogenic *Candida* spp. were found to be common in domestic washing machines in Hungary [9,40]. *Candida* species occurred frequently in such water-connected devices, mostly in hardly accessible parts of them. Further research is needed to survey the occurrence of the pathogenic *Candida* spp. in other water-connected devices of hospitals beyond BWDs.

### 3.1. Statistical Relationship of Fungi and Bacteria in BWDs

Fungi and bacteria often share common substrates, and their spatial proximity has led to antagonistic interactions in many environments, including aquatic ones [41]. This may explain our results of negative correlation between filamentous fungi and total HPC in water samples. If more time passed since the production date of the bottles, bacteria were more likely to proliferate in contrast to filamentous fungi. Furthermore, seasonal fluctuation was also observed, in April higher filamentous fungal counts have been experienced compared to July, when HPC results were significantly greater. Furthermore, in warmer months water consumption increases, therefore the connection between the higher quantity of ordered bottles and the lower number of yeasts in water might be also a seasonal consequence. Exposition of BWDs to sun—increasing water temperature—enhanced both HPC results, while exposition of water containers during storage increased only HPC at 37 °C. At the same time, filamentous fungal counts decreased significantly only during storage as a result of exposition to sunlight. It suggests that filamentous fungi and some bacteria proliferating at 37 °C are already present in the bottles. Previous studies on bottled water also reported about the presence of fungal contamination [3]. Furthermore, there is evidence of the bacterial contamination of the bottles of BWDs in Hungary as well. Thirteen bottles had been sampled, where two bottles contained *P. aeruginosa*, seven had high (>1000 CFU/mL) bacterial concentration at 22 °C, though only one had elevated counts (3800 CFU/mL) at 37 °C (unpublished data of Ágnes Sebestyén). On the other hand, several further parameters are connected mainly to operational and usage habits affect bacteria forming colonies at 22 °C—such as the type of institution, air filter in BWDs and the circle of users –, indicating that the contamination might originate partly from the devices’ inner environment. Furthermore, the HPC values at 37 °C were higher in case of BWDs operating in waiting areas of healthcare facilities, where presumably the device is accessed by more people with various hygienic habits. Filamentous fungal counts have decreased after maintenance or disinfection, while HPC values at 37 °C have increased.

Yamaguchi et al. [12] found positive correlation between HPC values and yeasts and HPC values and filamentous fungi, which is in contrast with our findings. Month of sampling (seasonal changing) and climate seem to have a major effect on the microbial contamination of stored bottles. Further conclusions cannot be drawn as we have no information about temperature and sampling season affecting microbial contamination of BWDs in Brazil.

Table S7 summarizes the related legislations of the European Union and Hungary for the tested microbial water quality parameters.

If the plate count in the bottle is assumed to be constant from bottling (which is generally not the case), values should not be higher than 10,000 CFU/mL and 2000 CFU/mL counts for HPC at 22 and 37 °C, respectively, based on the recommendation of Water Coolers Europe for BWDs. Compared to these values, 21.62% of water samples was compliant, 2.7% and 27.03% were non-compliant due to HPC at 22 °C and 37 °C, respectively, and in 48.65% both HPC results were non-compliant. None of the samples met the Hungarian empirical reference values used for drinking water.

### 3.2. Fungi in Water Samples, on Drip Trays and on Taps

According to our knowledge, this is the first study aiming to evaluate the fungal contamination and its causal factors in case of BWDs in Europe. Majority (86.8%) of the water samples were contaminated with fungi. In an unpublished survey of 33 BWDs in 2014, we found fungi in less (60.6%) water samples. The composition of fungi was also somewhat different (the most frequent fungi were *Cladosporium* (being present in 24% of the water samples and comprising 8% of the isolated fungal taxa) and *Penicillium* (18% of the water samples, 6% of the isolated fungal taxa), while *Botryotinia californica*, *Exophiala equina* and *Rhodotorula* spp. were also present). The difference in fungal contamination found in our previous and current study may be explained by the location of BWDs, as BWDs of the former sampling campaign were selected not only from healthcare institutions, but also from office buildings. Climatization and air filtration units are common in office buildings and provide low fungal concentration of the indoor air, because such devices filter out fungal spores from the air. In contrast, such mechanical ventilation systems are rare in healthcare facilities of Hungary [42,43]. Therefore, airborne contamination of BWDs seems to be more probable in healthcare facilities than in office buildings of Hungary. In the current study, 75.0% of fungal taxa isolated from water samples belonged to filamentous fungi, and 63.0% of them is characterized by slimy colonies and hydrophilic spores (gloiospores). This latter morphological character facilitates waterborne dispersal of spores [44]. Yeasts produce hydrophilic spores too. Therefore, most of the fungal taxa (72.2%) found in water samples of BWDs seem to be present due to a micro-environmental selection, favoring the waterborne dispersal. In contrast, hydrophilic taxa are less abundant in tray swab samples (52.6%). *Cladosporium* and *Rhodotorula* spp. dominate tray samples, which are also common in the air samples collected in Hungary (Donát Magyar, unpublished data). Their spores, settling from the air, often colonize surfaces covered with a thin film of water (e.g., condensate on window frames and drip trays, as in this case). Even though the contamination of BWDs from the air is probable, still remains speculative, therefore future studies should be designed to collect airborne fungi during water sampling.

Usually, pH is an important environmental parameter affecting fungal growth [45]. Counts of fungi in BWDs showed positive correlation with pH (range: 7.1–10.8). This finding is in contradiction to our previous results, where fungal counts of washing machines showed negative correlation with pH (range: 7–10.9) [9]. Low pH in washing machines is possibly due to the use of detergents, but such chemicals are not applied in BWDs. Even though water is a key environmental determinant in both devices, BWDs and washing machines offer a quite different environment for fungi.

Concentration of yeasts in water—similar to *P. aeruginosa*, capable to produce nitrite reductase enzyme [46]—correlated positively with nitrite ion concentration. Several species of the isolated yeasts or yeast-like genera are able to grow on nitrite (*Cryptococcus* spp., *Exophiala* spp., *Meyerozyma* spp., *Rhodotorula* spp., *Trichosporon* spp.) [47,48,49] or to reduce nitrate to nitrite or through nitrite to N_2_O or ammonia (*Acremonium* spp., *Aureobasidium* spp., *Blastobotrys* spp., *Cryptococcus* spp., *Exophiala* spp., *Geotrichum* spp., *Meyerozyma* spp., *Rhodotorula* spp.) [50,51,52,53,54,55,56]. Some genera might be able to oxidize nirite to nitrate (*Meyerozyma guilliermondii*, *Geotrichum* spp., *Rhodotorula* spp.) [57]. The possibility of a complex relationship between yeasts in water, *P. aerugimosa* and the nitrite ion concentration should be further investigated.

Counts of filamentous fungi and yeasts correlated, while Yamaguchi et al. [12] found no significant correlation between their occurrence in the bottled water samples [12]. These results also show the differences between the studies carried out in different climatic regions.

Air filters of BWDs are usually part of the water guard, directly contacting the bottles. These filters are able to keep out particles over 25 µm, while the size of fungal conidia common in the air may vary between 2–20 µm and bacterial cells fall between 0.5–1 µm, therefore contamination from the environment of BWDs is possible. The linkage between the presence of air filter and higher count results of HPC at 22 °C from water samples may be a result of airborne bacteria entering the device through the filter. In respect of the linkage between the presence of air filter and higher fungal contamination of drip trays the above-mentioned contamination pathway is less likely, as the air filter and drip tray are spatially separated. It is more likely that the drip tray is contaminated from the air. Not just the parameters of BWDs and usage habits, but also the exposition to sun during storage affected the filamentous fungal counts of drip trays. Therefore, we should assume that the contamination partly originates from the water itself and also from the air; and develops as biofilms with different species composition on surfaces exposed to air and water (drip trays and inner surfaces of the BWD, respectively).

Results of total fungi on the taps are mainly derived from the high concentration of yeasts. Their number decreased with higher alkalinity, total and temporary hardness. Several studies have shown that calcium increases surface attachment and biofilm production by many bacteria and fungi [58,59,60,61,62]. Divalent cations may play a role in the maintenance of biofilm structure as bridging agents, crosslinking the three-dimensional extracellular polymeric matrix [63,64], therefore hardening the formed biofilm [65]. Swab sampling has the disadvantage of providing information only about the immediate surface of a biofilm, unless the biofilm is soft enough to detach from its support during swabbing. In the latter case, yeast cells of deeper biofilm layers are also collected, which produces higher CFU values, therefore might explain the negative correlation of hardness with yeast numbers. Products of one manufacturer had significantly worse results, than two of the eight other manufacturers, indicating that yeast contamination of taps are dependent on the structure or material of the BWDs as well.

Counts of filamentous fungi on taps correlated with those counts of water samples, as well as with the expiration date of bottles. Therefore, filamentous fungi found on the taps might mainly originate from the bottles and inner pipelines which would contaminate the drained water (although some usage habits also affected the quality, but less significantly).

### 3.3. Health Considerations

Several opportunistic human pathogenic fungi were detected in the samples collected from BWDs, especially from water and tap surfaces, e.g., *Sarocladium kiliense* (previously isolated from clinical samples of vitreous fluid, cornea, sinus, bronchoalveolar lavage, esophagus, sputum, skin and toenail, etc. [66]), *Acremonium sclerotigenum*/*egyptiacum* (has been cultured from clinical samples of tracheal aspirate sinus, cerebrospinal fluid, bronchoalveolar lavage, sputum and toenail, etc. [22]), *Exophiala jeanselmei* var. *lecanii*-*corni* (has been linked to several systematic and localized cutaneous infections, known as causative agent of keratitis, pneumonia and other infections [67,68]), *Exophiala equina* (has been linked only to subcutaneous phaeohyphomycosis so far [69]), *Meyerozyma guilliermondii* (considered to have low pathogenicity, but has been found in respiratory, genital, soft tissue and skin specimens [70,71]), *Cystobasidium slooffiae* (has been linked to fungemia, endocarditis and meningitis [72]), *Aspergillus jensenii* (has been isolated from bronchoalveolar lavage, sputum and nail [73]), *Fusarium* (*Bisifusarium*) *biseptatum* (has been reported to cause trauma-related eye infection [31]), *Penicillium rubens* (has an ability to grow at 37 °C and was detected in several clinical samples from humans [74]), *Cladosporium halotolerans* (is not associated with human infection, but has been isolated from a number of clinical samples [75]). *Rhodotorula* species often colonize moist tubes, such as catheters (*R. mucilaginosa* is able to cause catheter-related fungemia) or hoses of aquarium filters. In our case, it is possible that these fungi colonized the plastic pipes in BWDs. Even though fungi are not considered as enteric pathogens, therefore their presence in drinking water is usually harmless, but patients after certain dental treatments might be at risk. Furthermore, these devices might act as sources of contamination accumulating opportunistic pathogenic species.

## 4. Materials and Methods

### 4.1. Study Design

A questionnaire was designed to provide information on the usage and disinfection habits of BWDs in Budapest, Hungary. For physical, chemical and biological observations 37 BWDs were sampled during the spring and summer of 2018. The sampled BWDs are located in medical institutions (7 in public hospitals, 14 in private hospitals, 7 in pharmacies and 10 in dentistries). The questionnaires were anonymously filled by the participants of the survey.

### 4.2. Questionnaire

The standardized questionnaire contained 17 questions, filled for each device by the operator/people in charge or other workers of the facilities on the usage and maintenance practice of the dispenser and the storage rooms of the device and the bottles. Furthermore, a record sheet has been filled, containing questions answerable by the samplers and on-site measurement results (temperature and relative humidity of the BWD’s and bottles’ storage rooms measured by Testo 174H portable temperature and humidity meter) (See Supplement 1. Questionnaire).

### 4.3. Sampling

Samples were collected from the cold water by opening the dispenser tap of the BWD according to EN ISO 19458. For microbiological and chemical analysis, 1 L cooled water samples were collected without purge in sterile glass bottles, while measuring the time needed to reach the volume. Furthermore, 250 mL cold water samples were also collected into acidified glass bottles for TOC analysis (rinsing the container 3 times before). For further microbiological analyses 500 mL heated water samples were collected into sterile glass bottles. Some additional cold water was taken to a small clean plastic glass to measure the temperature and pH on site and this process was repeated with hot water, where the device was able to provide it as well. Approximately one square cm of swab samples (by a sterile cotton tipped applicator with plastic shaft from Biolab Inc., St, Ontario, CA, USA) were collected from drip trays and taps, respectively, and spread onto malt extract agar (MEA) with 2% chloramphenicol on site. All samples have been transported in a cooler box (4–10 °C) to the National Public Health Center within 1–3 h, where they were uniformly stored at 4 °C until processing. The collected water samples were divided for the different analyses in lab conditions. TOC samples were acidified to pH < 2.

### 4.4. Physical Analyses

The following physical parameters were tested: temperature (°C, on site by using Testo 206-pH1, Testo INC, Lenzkirch, Germany), taste and odor (based on the experience of the operator of the BWD), conductivity (WTW inoLab Cond Level 2 was used with a WTW TetraCon 325 cell, Xylem INC, Global), volume flow (on site, time necessary to draw 1 L of cold water sample from the BWD, expressed in seconds/L).

### 4.5. Chemical Analyses

The following parameters have been measured: pH (on site by a Testo 206-pH1, Testo INC, Lenzkirch, Germany), alkalinity (MSZ 448-11:1986), total hardness (MSZ 448-21:1986), ammonium (MSZ ISO 7150-1:1992), nitrite concentration (MSZ 1484-13:2009), total organic carbon (TOC/TNb elementar vario TOC cube, Elementar Analysensysteme GmbH, Langenselbold, Germany).

### 4.6. Microbiology

Heterotrophic plate counts (HPC) at 22 °C and 37 °C were determined according to the MSZ EN ISO 8199:2005 [76] and detection and quantification of *P. aeruginosa* were performed according to the MSZ EN ISO 16266:2006 [77] standards.

For the characterization of fungal contamination of the drinking water, 100 mL water sample was concentrated on cellulose nitrate membrane filter (pore diameter: 0.45 µm) by using a two-stage vacuum pump. Filters were placed on malt extract agar (MEA) with 2% chloramphenicol. Plates were incubated at 25 °C for 5 days. The incubation temperature is based on the usual temperature of the environment by the devices, while the number of days were decreased to 5 days (the Swedish legislation use 7 days—Babic et al. 2017), as several samples were highly contaminated. Following the incubation, fungal colonies have been counted and common (>4 CFU/100mL) morphotypes were selected for isolation to potato dextrose agars (Biolab Inc.) containing 0.02% streptomycin (Sigma-Aldrich Chemie Gmbh., St. Louis, MO, USA). Isolated and purified strains were identified at the genus level based on their morphological characteristics examined by a Carl Zeiss Jenaval light microscope at 300× magnification. The most common species have been identified at species level based on molecular characterization of the internal transcribed spacer region of the ribosomal RNA gene cluster enclosed by the ITS1F Forward (5′-CTT GGT CAT TTA GAG GAA GTA A-3′) and ITS4 Reverse primers (5′-TCC TCC GCT TAT TGA TAT GC-3′) [78,79]. For subsets of the isolates a fragment of the translation elongation factor (*tef*) 1α gene was also amplified with primers EF1-728F (5′-CATCGAGAAGTTCGAGAAGG) and TEF-LLErev (5′-AACTTGCAGGCAATGTGG) following the protocol described by Hatvani et al. [80]. Sanger-sequencing of the amplicons was performed on ABI 3130 Genetic Analyzer (Applied Biosystems, USA) at Institute of Aquaculture and Environmental Safety, Gödöllő and 3500 Genetic Analyzer (Life Technologies) at BayGen, Szeged. Species identification was performed by nucleotide-nucleotide BLAST analysis [81] at the website of the National Center of Biotechnology Information (NCBI, www.ncbi.nlm.nih.gov, accessed on 9 July 2021).

### 4.7. Statistical Analysis

In many samples, the HPC values exceeded the upper limit of quantification (10,000 CFU/mL), thus for the statistical analyses an ordinal scale was generated by categorizing the data into groups of 500 CFU/mL increments, where the last group contains the samples exceeding 9500 CFU/mL.

For the determination of connections between parameters, we used Pearson correlation with Benjamini-Hochberg correction for the numerical variables (where appropriate, we applied data transformation technics), and Pairwise Wilcoxon-Mann-Whitney test also with Benjamini-Hochberg correction for factors, which are less sensitive for unbalanced data.

All statistical calculations have been performed in R-statistics (www.r-project.org, accessed on 9 July 2021).

## 5. Conclusions

The modern lifestyle and the change of water consumption habits, including the use of new water-containing household devices such as bottled water dispensers require constant improvement of regulations, water purification and monitoring practices. The present study revealed that bottled water dispensers provide excellent habitat for manifold microorganisms. According to our two previous works [3,82] and the present one, it can be concluded that a high percentage of BWDs are contaminated with bacteria, filamentous fungi and yeasts. Such microorganisms are able to proliferate in the bottled water, disperse inside the BWD devices and colonize their surfaces, which may pose a health risk especially for immunocompromised people visiting healthcare institutions. Stagnant water in the bottle and the device is disadvantageous. It is advisable to optimize the storage time, as well as the time while the bottle is in use. Most optimal if the bottle is replaced after 2–3 days, regardless of how much water is consumed. Regular and professional cleaning of the devices is recommended. Considering the number and variety of identified pathogens, operation of BWDs in high-risk areas such as health care facilities should be a concern, and other means of providing staff and patients with safe drinking water should be preferred. Our study (conducted in a country of well-regulated water hygiene system) draws attention to the need of considering the risks associated with fungi in BWDs in other parts of the world as well.

## Figures and Tables

**Table 1:** Significant Pearson correlations between concentration of fungi and the parameters of water derived from BWDs.

Pearson Correlation with Benjamini-Hochberg Correction				
Parameter A	Parameter B [CFU/100 mL]	*p*	R	Corrected *p*
pH	Total Fungi ^a^	0.0005	0.41	0.007
	* Yeasts ^a^	0.0054	0.33	0.0252
	* Filamentous Fungi ^a^	0.02	0.28	0.04
Time since the last disinfection or maintenance (days)	Total Fungi ^a^	0.0064	0.62	0.0448
	* Filamentous Fungi ^a^	0.0056	0.62	0.0196
Temperature of water [°C]	Total Fungi ^a^	0.0079	−0.32	0.0369
	* Filamentous Fungi ^a^	0.0086	−0.32	0.0241

^a^: logarithmized data. *: subcomponent variables contributing to the mean variable (Total Fungi).

**Table 2:** Fungal species detected in water and swab samples (>4 morphologically identical CFU) collected from bottled water dispensers in medical institutions.

Identification	ID	Type	Identification Type	GenBank Accession Number of:		Relative Frequency in the Sample	Reference
				ITS	*tef1*α		
*Acremonium egyptiacum*	B2131C	cold water	Molecular	MT320780	-	0.27	[22] Perdomo et al., 2011 (FN706550)
*Acremonium sclerotigenum*	B2078	cold water	Molecular	-	MZ190340		-
*Acremonium sclerotigenum*	B2080	cold water	Molecular	MT320766	-	0.97	[22] (NR_149332)
*Acremonium sclerotigenum*	B2131B	cold water	Molecular	MT320775	MZ190338	0.2	[22] (NR_149332)
*Acremonium sclerotigenum*	B2081	hot water	Molecular	MT320773	-	0.98	[22] (NR_149332)
*Acremonium sclerotigenum*	B03TB	tray swab	Molecular	-	MZ190332	-	-
*Acremonium* sp.	B20A	cold water	Morphological	-	-	0.33	-
*Acremonium* sp.	B2242B	cold water	Morphological	-	-	0.27	-
*Acremonium* sp.	B4003B	cold water	Morphological	-	-	0.02	-
*Acremonium* sp.	B4005A	cold water	Morphological	-	-	1	-
*Acremonium* sp.	B4402A	cold water	Morphological	-	-	0.94	-
*Acremonium* sp.	B11CB	tap swab	Morphological	-	-	-	-
*Acremonium* sp.	B22TD	tray swab	Morphological	-	-	-	-
*Acremonium* sp.	B33TA	tray swab	Morphological	-	-	-	-
*Aspergillus jensenii*	B19CSA	tap swab	Molecular	MT320779	-	-	[23] (NR_135444)
*Aspergillus steynii*	B2132B	cold water	Molecular	-	MZ190334	0.02	-
*Aspergillus* sp.	B2132C	cold water	Morphological	-	-	0.53	-
*Aspergillus* sp.	B2245D	cold water	Morphological	-		0.04	-
*Aureobasidium* sp.	B2245G	cold water	Morphological	-	-		-
*Aureobasidium* sp.	B14TD	tray swab	Molecular	-	MZ190333	-	-
*Blastobotrys* sp.	B4008C	cold water	Morphological	-	-	0.08	-
*Cadophora malorum*	B2130D	cold water	Molecular	MT320771	-	0.07	[24] (NR_145268)
*Candida oleophila*	B37TC	tray swab	Molecular	MT320774	-	-	[25] (NR_155224)
Chaetothyriales sp.	B2244E	cold water	Molecular	-	MZ190326	0.13	-
*Cladosporium cladosporioides*	B16HA	cold water	Morphological	-	-	0.29	-
*Cladosporium cladosporioides*	B19TA	tray swab	Morphological	-		-	-
*Cladosporium halotolerans*	B2130A	cold water	Molecular	MT320762	-	0.79	[26] (NR_119605/DQ780364)
*Cladosporium* sp.	B2132F	cold water	Morphological	-	-	0.07	-
*Cladosporium* sp.	B05TB	tray swab	Morphological	-	-	-	-
*Cladosporium* sp.	B09TB	tray swab	Morphological	-	-	-	-
*Cladosporium* sp.	B09TC	tray swab	Morphological	-	-	-	-
*Cladosporium* sp.	B19TB	tray swab	Morphological	-	-	-	-
*Cladosporium* sp.	B23TC	tray swab	Morphological	-	-	-	-
*Cladosporium* sp.	B30T	tray swab	Morphological	-	-	-	-
*Cryptococcus* sp.	B4009A	cold water	Morphological	-	-	0.93	-
*Cylindrocarpon* sp.	B4390B	cold water	Morphological	-	-	0.47	-
*Cystobasidium minutum*	B15TB	tray swab	Molecular	-	MZ190335	-	-
*Cystobasidium slooffiae*	B2242A	cold water	Molecular	MT320776	-	0.04	[27] (NR_103568/AF444627)
*Cystobasidium slooffiae*	B14TC	tray swab	Molecular	MT320767	-	-	[27] (NR_103568/AF444627)
*Cystobasidium slooffiae*	B2130B	cold water	Molecular	MW166334	-	0.13	[27] (NR_103568/AF444627)
*Didymella protuberans*	B2245A	cold water	Molecular	MT320764	-	0.02	[28] (NR_135993/GU237853)
*Exophiala alcalophila*	B0708C	tap swab	Molecular	MT320777	-	-	[29] (NR_111624)
*Exophiala equina*	B4003A	cold water	Molecular	MT320769	-	0.7	[29] (NR_111627)
*Exophiala lecanii-corni*	B2242C	cold water	Molecular	MT320770	MZ190330	0.63	[30] (NR_145351/AY857528)
*Exophiala lecanii-corni*	B4003D	cold water	Molecular	MT320768	-	0.14	[30] (NR_145351/AY857528)
*Exophiala* sp.	B11CA	tap swab	Molecular	-	MZ190337	-	-
*Fusarium* *dimerum*	B02TC	tray swab	Molecular	MT320778	-	-	[31] (NR_137706)
*Fusarium* sp.	B2132G	cold water	Morphological	-	-	0.16	-
*Geotrichum* sp.	B2244B	cold water	Morphological	-	-	0.07	-
*Gliomastix polychroma*	B2245B	cold water	Molecular	MT320759	-	0.02	[32] (NR_119408)
*Gliomastix* sp.	B33TB	tray swab	Morphological	-	-	-	-
*Meyerozyma guilliermondii*	B02TA	tray swab	Molecular	MT320761	-	-	[25] (KY104252)
*Oidiodendron* sp.	B31TA	tray swab	Morphological	-	-	-	-
*Paecilomyces* sp.	B19CSA	tap swab	Morphological	-	-	-	-
*Penicillium* *chrysogenum*	B2132A	cold water	Molecular	MT320763	MZ190336	0.05	[33] (NR_111815)
*Penicillium* sp.	B4008A	cold water	Morphological	-	-	0.82	-
*Penicillium* sp.	B15CSA	tap swab	Morphological	-	-	-	-
*Penicillium* sp.	B20TA	tray swab	Morphological	-	-	-	-
*Penicillium* sp.	B23TA	tray swab	Morphological	-	-	-	-
*Penicillium* sp.	B23TB	tray swab	Morphological	-	-	-	-
*Phaeoramularia* sp.	B4397MA	hot water	Morphological	-	-	0.5	-
*Purpureocillium lilacinum*	B32TA	tray swab	Molecular	-	MZ190328	-	-
*Purpureocillium lilacinum*	B2130C	cold water	Molecular	-	MZ190339		-
*Pyricularia* sp.	B34CSA	tap swab	Morphological	-	-	-	-
*Rhodotorula* sp.	B2245E	cold water	Morphological	-	-	0.14	-
*Rhodotorula* sp.	B2245F	cold water	Morphological	-	-	0.07	-
*Rhodotorula* sp.	B02TB	tray swab	Morphological	-	-	-	-
*Rhodotorula* sp.	B0708T	tray swab	Morphological	-	-	-	-
*Rhodotorula* sp.	B09TA	tray swab	Morphological	-	-	-	-
*Rhodotorula* sp.	B14TB	tray swab	Morphological	-	-	-	-
*Rhodotorula* sp.	B15TC	tray swab	Morphological	-	-	-	-
*Rhodotorula* sp.	B28TA	tray swab	Morphological	-	-	-	-
*Sarocladium kiliense*	B2244D	cold water	Molecular	-	MZ190325	0.36	-
*Scopulariopsis* sp.	B22TE	tray swab	Morphological	-	-	-	-
*Simplicillium cylindrosporum*	B2132E	cold water	Molecular	MT320760	-	0.33	[34] (NR_111023)
*Simplicillium* *lanosoniveum*	B4009B	cold water	Molecular	MT320765	MZ190331	0.05	[34] (NR_111025)
*Simplicillium minatense*	B2132D	cold water	Molecular	-	MZ190329	0.27	-
*Simplicillium minatense*	B22TA	tray swab	Molecular	-	MZ190327	-	-
*Simplicillium* sp.	B4008B	cold water	Morphological	-	-	0.003	-
*Talaromyces* sp.	B23TD	tray swab	Morphological	-	-	-	-
*Trichosporon* sp.	B37TA	tray swab	Morphological	-	-	-	-
Unidentified filamentous sp.	B09TD	tray swab	Morphological	-	-	-	-
Unidentified filamentous sp.	B2131A	cold water	Morphological	-	-	0.1	-
Unidentified filamentous sp.	B2244A	cold water	Morphological	-	-	0.07	-
Unidentified filamentous sp.	B4007A	cold water	Morphological	-	-	0.08	-
Unidentified filamentous sp.	B4390A	cold water	Morphological	-	-	0.50	-
Unidentified filamentous sp.	B14MA	hot water	Morphological	-	-	0.8	-
Unidentified yeast sp.	B03TA	tray swab	Morphological	-	-	-	-
Unidentified yeast sp.	B05TA	tray swab	Morphological	-	-	-	-

## Data Availability

The data presented in this study are available in the Supplementary Materials.

## Supplementary Materials

The following are available online at https://www.mdpi.com/article/10.3390/pathogens10070871/s1, Supplement 1: Questionnaire, Figure S1: Correlation of the heterotrophic plate counts (HPC) of bacteria and the TOC results of water samples derived from bottled water dispensers placed in medical institutions, Figure S2: Tendencies of counts of heterotrophic plate counts (HPC) of bacteria and filamentous fungi in bottled water dispensers placed in medical institutions, as the water bottles expire, Figure S3: Correlation of the HPC at 22 °C and filamentous fungal counts (y = −657.1 * x + 8679.3, *p* = 0.02982, R = −0.36) in bottled water dispensers placed in medical institutions, Figure S4: Heterotrophic plate counts (HPC) of bacteria and filamentous fungi in different sampling months of the bottled water dispensers placed in medical institutions, Figure S5: Heterotrophic plate counts (HPC) of bacteria in water samples collected from bottled water dispensers placed in distinct medical institutions, Figure S6: Filamentous fungi on drip tray and volume flow results of the water derived from bottled water dispensers in medical institutions (y = 0.1 * x + −1.1, *p* = 0.00134, R = 0.56), Table S1: Statistical description of numerical variables from the survey of bottled water dispensers in medical institutions, Table S2: Statistical description of measured numerical variables from the survey of bottled water dispensers in medical institutions. *: Subcomponent referred in the study. NA: Number of samples, for which data was not available, Table S3: Significant relationships of filamentous fungal concentrations in water samples [CFU/100 mL] derived from bottled water dispensers in medical institutions. a: logarithmized data, Table S4: Significant relationships of yeast concentration in water samples [CFU/100 mL] derived from bottled water dispensers in medical institutions. a: logarithmized data, Table S5: Significant relationships of fungi on drip trays of bottled water dispensers in medical institutions. a: logarithmized data, Table S6: Connections of fungal counts of swab samples collected from the taps of bottled water dispensers to other parameters in medical institutions. a: logarithmized data, *: subcomponent variables contributing to the mean variable (Total Fungi).
