# Supplementary material for: Mycological Investigation of Bottled Water Dispensers in Healthcare Facilities
Source: Pathogens. 2021 Jul 10;10(7):871. doi: 10.3390/pathogens10070871 (PMC8308914; doi:10.3390/pathogens10070871)
Supplement: Supplementary file 1 [file pathogens-10-00871-s001.zip › Supplement 1.Questionnaire.pdf]

Sample ID:..

Questionnaire for the operation of Bottled Water Dispensers (BWDs)

Please underline the correct answer (there may be more if necessary) and answer the questions

I. How often do you drink water from the BWD?

1. daily **2.** 2-3 times a week **3.** weekly

4. 2-3 weeks **5.** less often **6.** never

II. Have you or your colleague noticed an unusual taste when consuming water?

1. yes **2.** no

III. Have you or your colleague noticed an unusual odour when consuming water?

1. yes **2.** no

IV. Have you or your colleague experienced any other problems with water?

1. yes **2.** no

a., if yes, what?

…………………………………………………………………………………………………..

V. People who visiting the BWD's room:

1. workers **2.** customers **3.** patients **4.**other: ……………….

VI. Frequency of ventillation of the BWD's room:

1. many times a day **2.** once a day **3.** several times a week

4. less often **5.** never

VII. People who using the BWD:

1. workers **2.** customers **3.** patients **4.**other: ……………….

VIII. Who carries out the disinfection?

1. service **2.** workers **3.** nobody

IX. Frequency of disinfection:

1. more frequent than monthly **2.** 1-2 per month **3.** 3-4 per month

4. 5-6 per month **5.** yearly **6.** less often **7.** never

Sample ID:...

X. Date of the last disinfection: …………………………………………………………...

XI. Last date of other maintenance: ……………………………………………...

XII. Does bottled water always come from the same distributor?

1. yes **2.** no

a., if not, other brands:
……………………………………………………………………………………………………
……………………………………………………………………………………………………
……………………………………………………………………………………...

XIII. Date of last bottle change: ………………………………………………

XIV. Typically, how often are bottles ordered?

1. weekly **2.** monthly **3.** 2-3 times a month

4. every half a year **5.** yearly **6.** less often

XV. Typically, how many bottles are ordered?

1. 1-5 pcs **2.** 6-10 pcs **3.** 11-20 pcs **4.** more

XVI. Typically, how long are the unopened bottles stored?

1. for up to 2 weeks **2.** for up to 2-4 weeks

3. for up to 2-3 months **4.** for up to half a year

5. there was an example that it was stored for more than 6 months

XVII. Typically, how often is the bottle storage room ventilated?

1. many times a day **2.** once a day **3.** several times a week

4. less often **5.** never

Thank you for completing the questionnaire!

Sample ID:...

<u>To be completed by the examiner:</u>

<u>I.</u> Sample ID: ………………………………………………………………...

<u>II.</u> Samplers: ....

<u>III.</u> Date of sampling: ………………………………………………………….

<u>IV.</u> Name and address of institution:
……………………………………………………………………………………………………
……………………………………………………………………………………………………
……………………………………………………………………………………...

<u>V.</u> Type of the institution:

1. hospital **2.** pharmacy **3.** laboratory **4.** other: ……………………...

<u>VI.</u> Name of the contact person: ……………………………………………………………..

<u>VII.</u> Contact person's phone number or email address:
……………………………………………………………………………...

<u>VIII.</u> Type of bottled water dispenser room:

1. kitchen, dining room **2.** customer area, waiting room

3. corridor **4.** other: ……………………………………...

<u>IX.</u> Is there mold in the BWD's room?

1. yes **2.** no

<u>X.</u> Is there ventilation in the BWD's room (functioning ventilation system, air intake slot, conventional (non-plastic framed) window or plastic framed window with air inlet)?

1. yes **2.** no

<u>XI.</u> Is there a hood in the BWD's room?

1. yes **2.** no

<u>XII.</u> Measured in the BWD's room:

a., temperature (°C): ……………………………………….

b., humidity (RH%): ……………………………………...

<u>XIII.</u> Can the BWD be exposed to solar radiation?

Sample ID:...

1. yes **2.** no

<u>XIV.</u> BWD type, manufacturer:
…………………………………………………………………………………………………
…………………………………………………………………………………………………
……………………………………………………………………………………

<u>XV.</u> OTH license: ………………………………………………………………….

<u>XVI.</u> Is the user manual available?

1. yes **2.** no

a., If yes, what are the disinfection requirements? (photo)
…………………………………………………………………………………………………
…………………………………………
…………………………………………………………………………………………………
…………………………………………
…………………………………………………………………………………………………
………………………………………….

<u>XVII.</u> Does the BWD have a buffer tank?

1. yes **2.** no

<u>XVIII.</u> Can carbonated water be made with the BWD?

1. yes **2.** no

<u>XIX.</u> Distributor of bottled water:
……………………………………………………………………………………………..

<u>XX.</u> The amount of water in the bottle at the start of sampling:
………………………………………………………………………….

<u>XXI.</u> Bottle expiration date:
…………………………………………………………………………………………………
…………

<u>XXII.</u> Bottle material:
…………………………………………………………………………………………………
……………….

<u>XXIII.</u> Ballonok tárolására szolgáló helyiségnek jellege:

1. kitchen, dining room **2.** customer area, waiting room

3. corridor **4.** store room **5.** other:
……………………………………...

XXIV. Does the bottle storage room have a window?

1. yes **2.** no

XXV. Is there room for ventilation in the bottle storage room?

1. yes **2.** no

XXVI. Is there a dehumidifier in the bottle storage room?

1. yes **2.** no

XXVII. Measured in the bottle storage room:

a., temperature (°C): ……………………………………….

b., humidity (RH%): ……………………………………...

XXVIII. Can bottles be exposed to solar radiation?

1. yes **2.** no

XXIX. Other comments:
…………………………………………………………………………………………………
…………………………………………………………………………………………………
…………………………………………………………………………………………………
…………………………………………………………………………………………………
…………………………………
