# Supplementary material for: Mycological Investigation of Bottled Water Dispensers in Healthcare Facilities
Source: Pathogens. 2021 Jul 10;10(7):871. doi: 10.3390/pathogens10070871 (PMC8308914; doi:10.3390/pathogens10070871)
Supplement: Supplementary file 1 [file pathogens-10-00871-s001.zip › Supplementary captions.pdf]

Supplement 1: Questionnaire

Supplementary tables:

Table S.1. Statistical description of numerical variables from the survey of bottled water dispensers in medical institutions

Table S.2. Statistical description of measured numerical variables from the survey of bottled water dispensers in medical institutions. *: Subcomponent referred in the study. NA: Number of samples, for which data was not available

Table S.3. Significant relationships of filamentous fungal concentrations in water samples [CFU/100 ml] derived from bottled water dispensers in medical institutions. a: logarithmized data.

Table S.4. Significant relationships of yeast concentration in water samples [CFU/100 ml] derived from bottled water dispensers in medical institutions. a: logarithmized data.

Table S.5. Significant relationships of fungi on drip trays of bottled water dispensers in medical institutions. a: logarithmized data.

Table S.6. Connections of fungal counts of swab samples collected from the taps of bottled water dispensers to other parameters in medical institutions. a: logarithmized data, *: subcomponent variables contributing to the mean variable (Total Fungi)

Supplementary figures:

Figure S.1. Correlation of the heterotrophic plate counts (HPC) of bacteria and the TOC results of water samples derived from bottled water dispensers placed in medical institutions.

Figure S.2. Tendencies of counts of heterotrophic plate counts (HPC) of bacteria and filamentous fungi in bottled water dispensers placed in medical institutions, as the water bottles expire

Figure S.3. Correlation of the HPC at 22 °C and filamentous fungal counts ($y=-657.1*x+8679.3$, $p=0.02982$, $R=-0.36$) in bottled water dispensers placed in medical institutions.

Figure S.4. Heterotrophic plate counts (HPC) of bacteria and filamentous fungi in different sampling months of the bottled water dispensers placed in medical institutions.

Figure S.5. Heterotrophic plate counts (HPC) of bacteria in water samples collected from bottled water dispensers placed in distinct medical institutions.

Figure S.6. Filamentous fungi on drip tray and volume flow results of the water derived from bottled water dispensers in medical institutions ($y=0.1*x+-1.1$, $p=0.00134$, $R=0.56$)

Raw data file added (raw_data_bwd.tsv).
