# Supplementary material for: Mycological Investigation of Bottled Water Dispensers in Healthcare Facilities
Source: Pathogens. 2021 Jul 10;10(7):871. doi: 10.3390/pathogens10070871 (PMC8308914; doi:10.3390/pathogens10070871)
Supplement: Supplementary file 1 [file pathogens-10-00871-s001.zip › Table S7.pdf]

**Table S.7** Legislation for the tested bacterial water quality parameters

| Legislation | Heterotrophic plate count at 22 °C | Heterotrophic plate count at 37 °C | *Pseudomonas aeruginosa* |
|---|---|---|---|
| Guidelines for Drinking water: European Union and Hungary (98/83/EC; 201/2001. (X.25.) Gov.Reg.) | No abnormal change | | 0 CFU/100 ml |
| Parametric values for Bottled water (at the point of bottling): European Union and Hungary (98/83/EC; 201/2001. (X.25.) Gov.Reg.) | 100 CFU/ml | 20 CFU/ml | 0 CFU/250 ml |
| Recommendations of Water Coolers Europe for Bottled Water Dispensers[1] (Watercoolers Europe, 2016) | Maximum 2 log increments compared to the entry point | | - |
| Empirical reference values used in Budapest, Hungary for drinking water[2] | 400 CFU/ml | 80 CFU/ml | - |

[1]Values calculated based on the parametric values of the European Union for bottled water
[2]On the basis of oral communication by Á. Sebestyén

## References


Governmental Decree No. 201 of 2001 (X.25.) Korm. on quality standards for potable water and on rules of quality control. Hungary

Watercoolers Europe (2016) Guidelines for Good Hygienic Practice For Distributors and Operators of Plumbed-in (POU – Point of Use) Water Coolers, "European Commission," 5. Sept. 2016. [Online]. Available: https://ec.europa.eu/food/system/files/2017-02/biosafety_fh_guidance_guidelines_water_coolers.pdf [Accessed 20. 06. 2021.]
