# Supplementary figures and images for: Mycological Investigation of Bottled Water Dispensers in Healthcare Facilities
Source: Pathogens. 2021 Jul 10;10(7):871. doi: 10.3390/pathogens10070871 (PMC8308914; doi:10.3390/pathogens10070871)

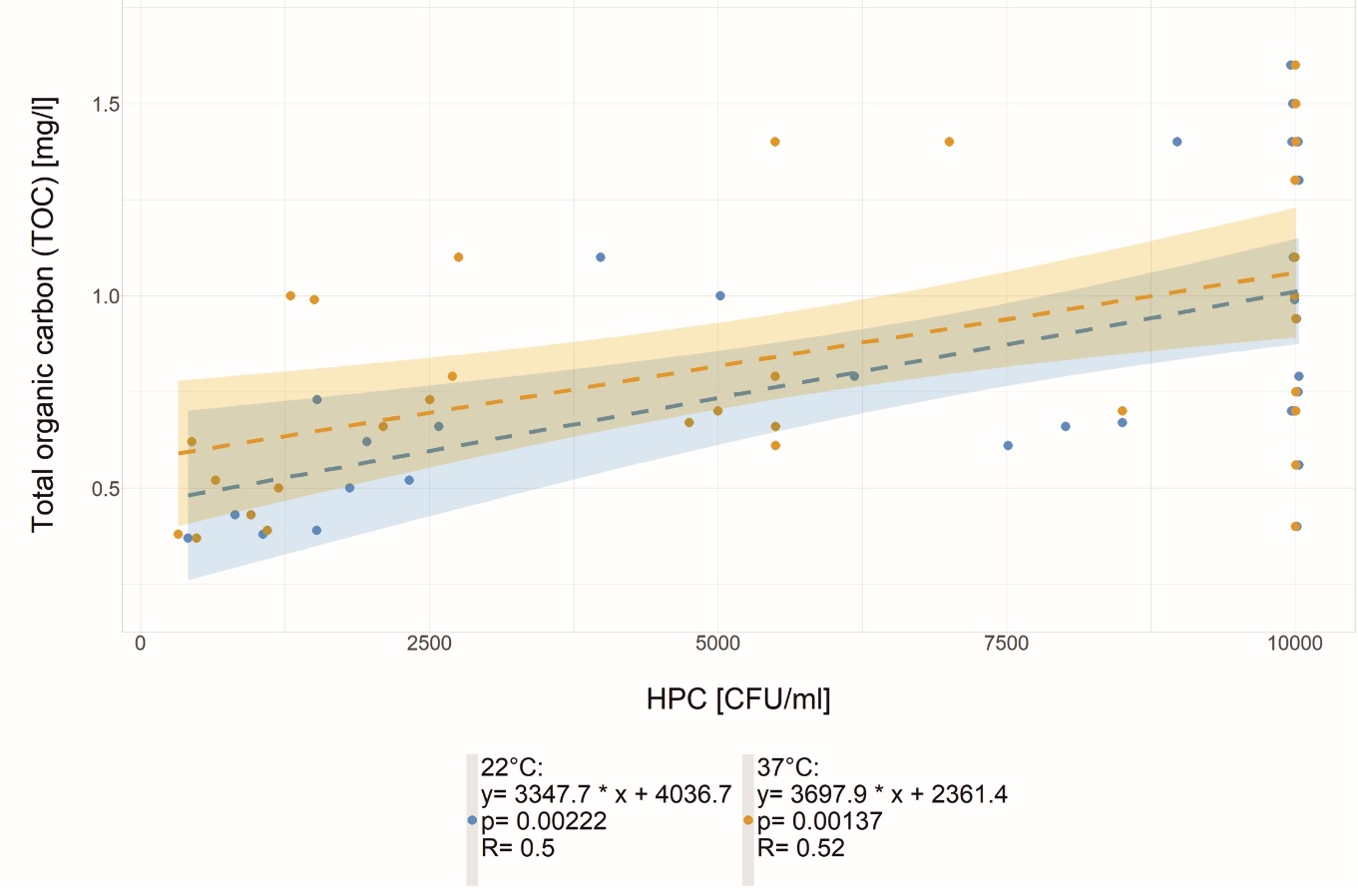

Supplement: Supplementary file 1 [file pathogens-10-00871-s001.zip › Figure S1.jpg]

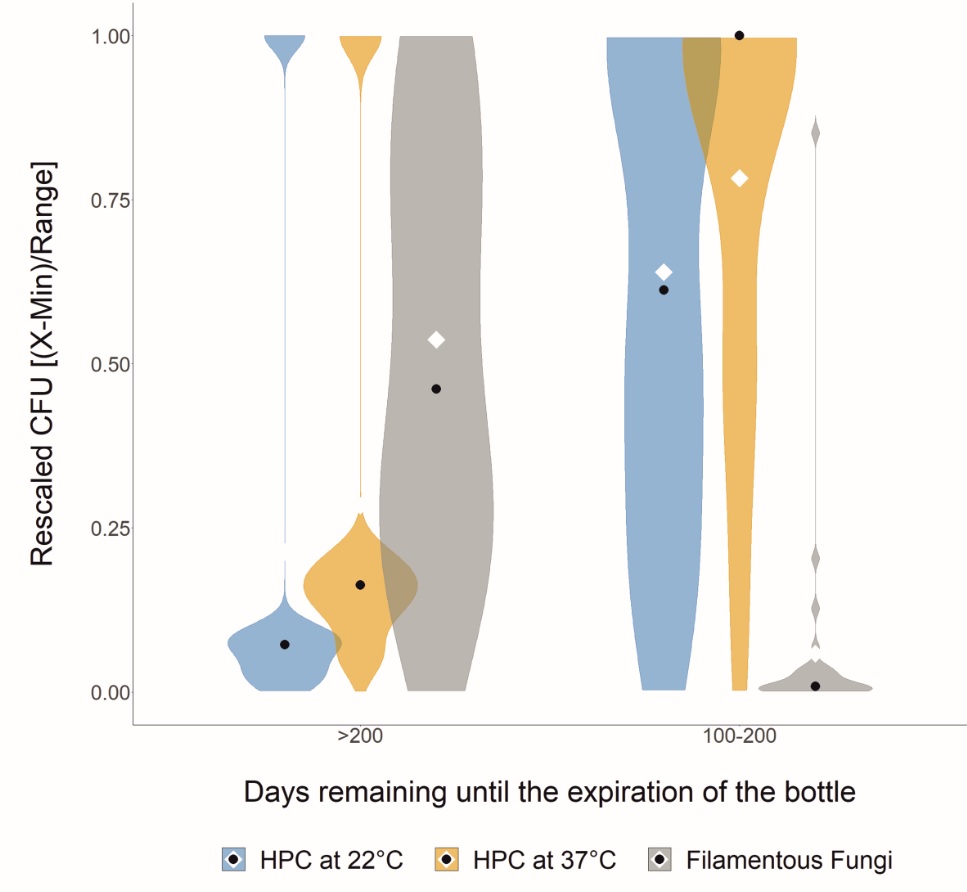

Supplement: Supplementary file 1 [file pathogens-10-00871-s001.zip › Figure S2.jpg]

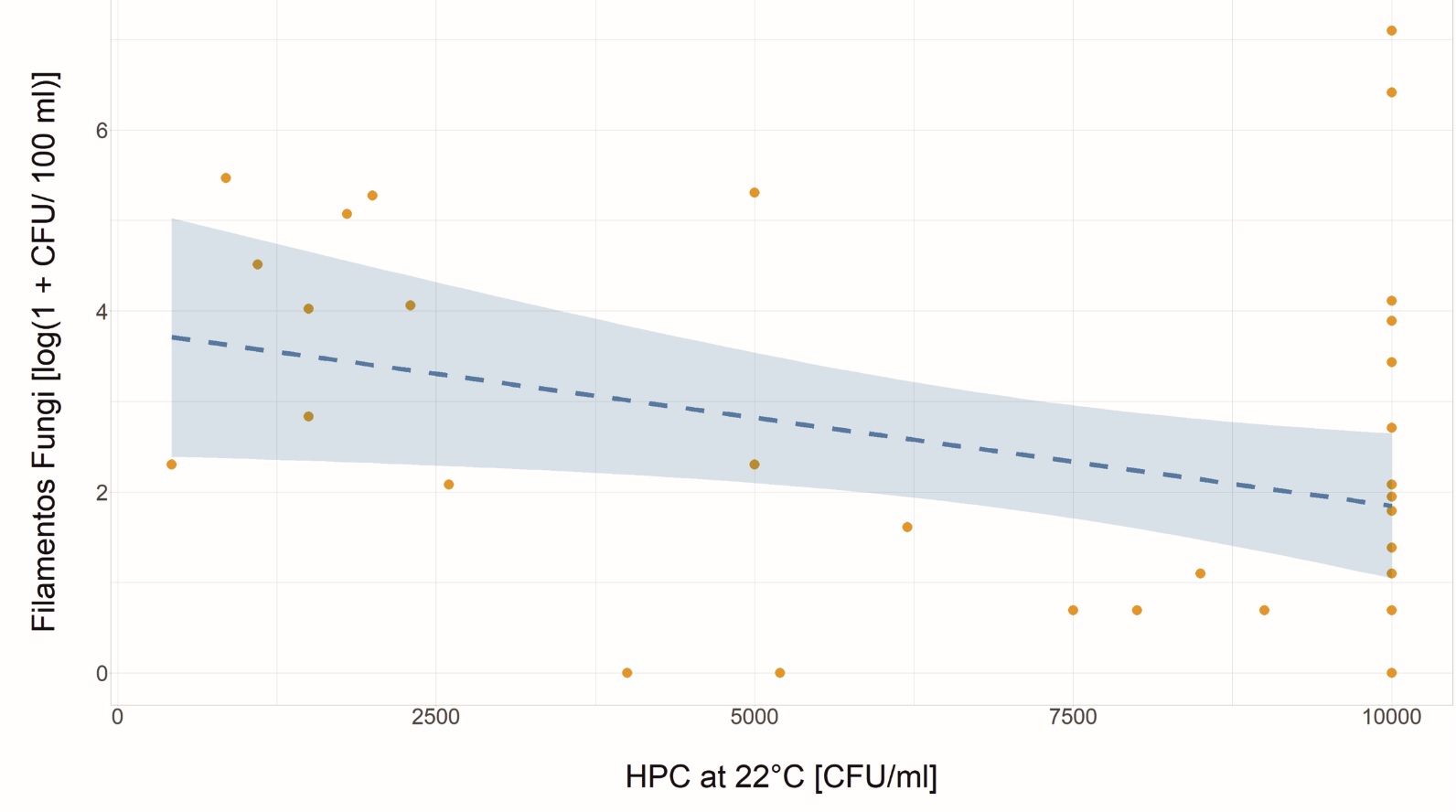

Supplement: Supplementary file 1 [file pathogens-10-00871-s001.zip › Figure S3.jpg]

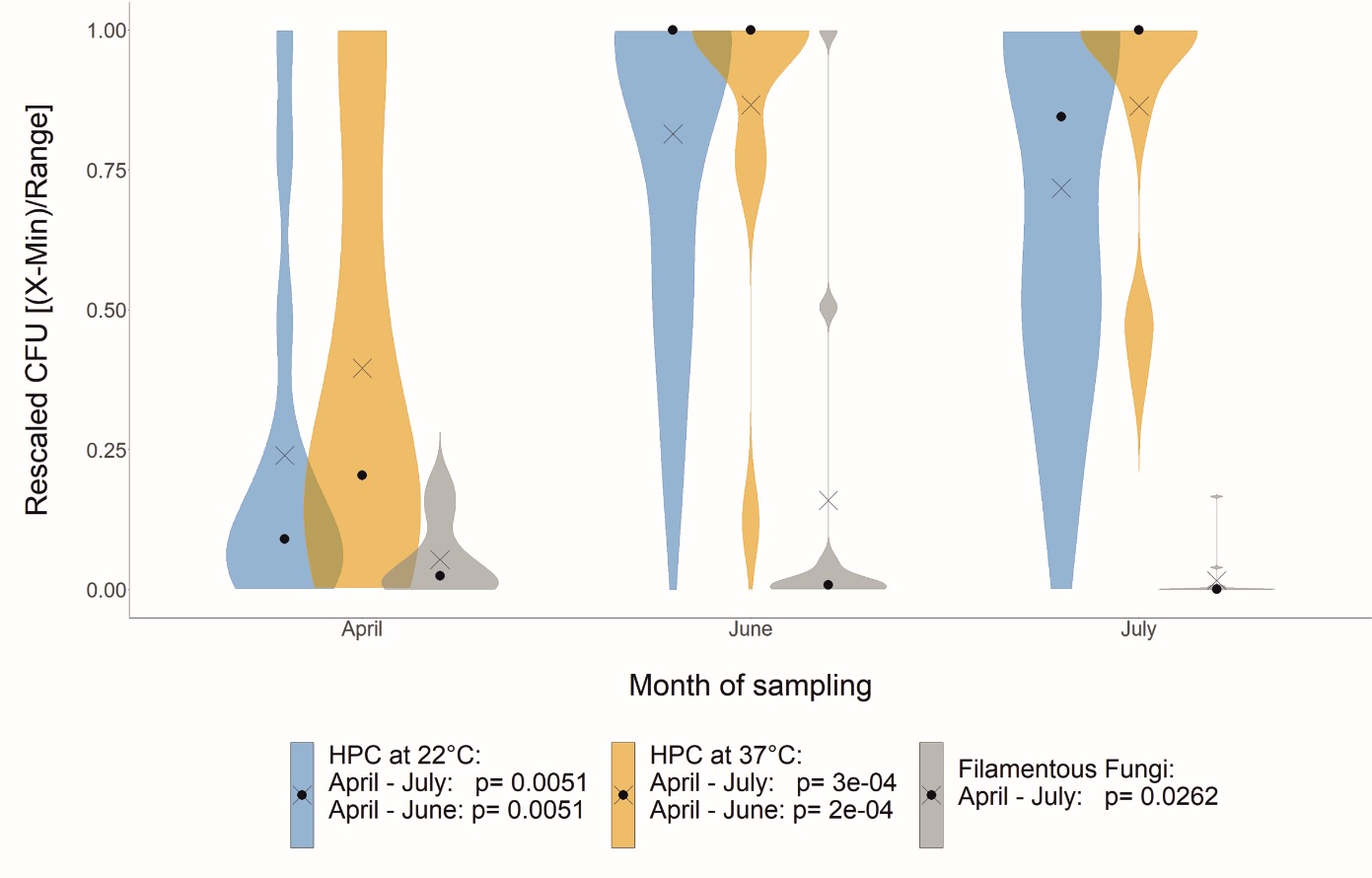

Supplement: Supplementary file 1 [file pathogens-10-00871-s001.zip › Figure S4.jpg]

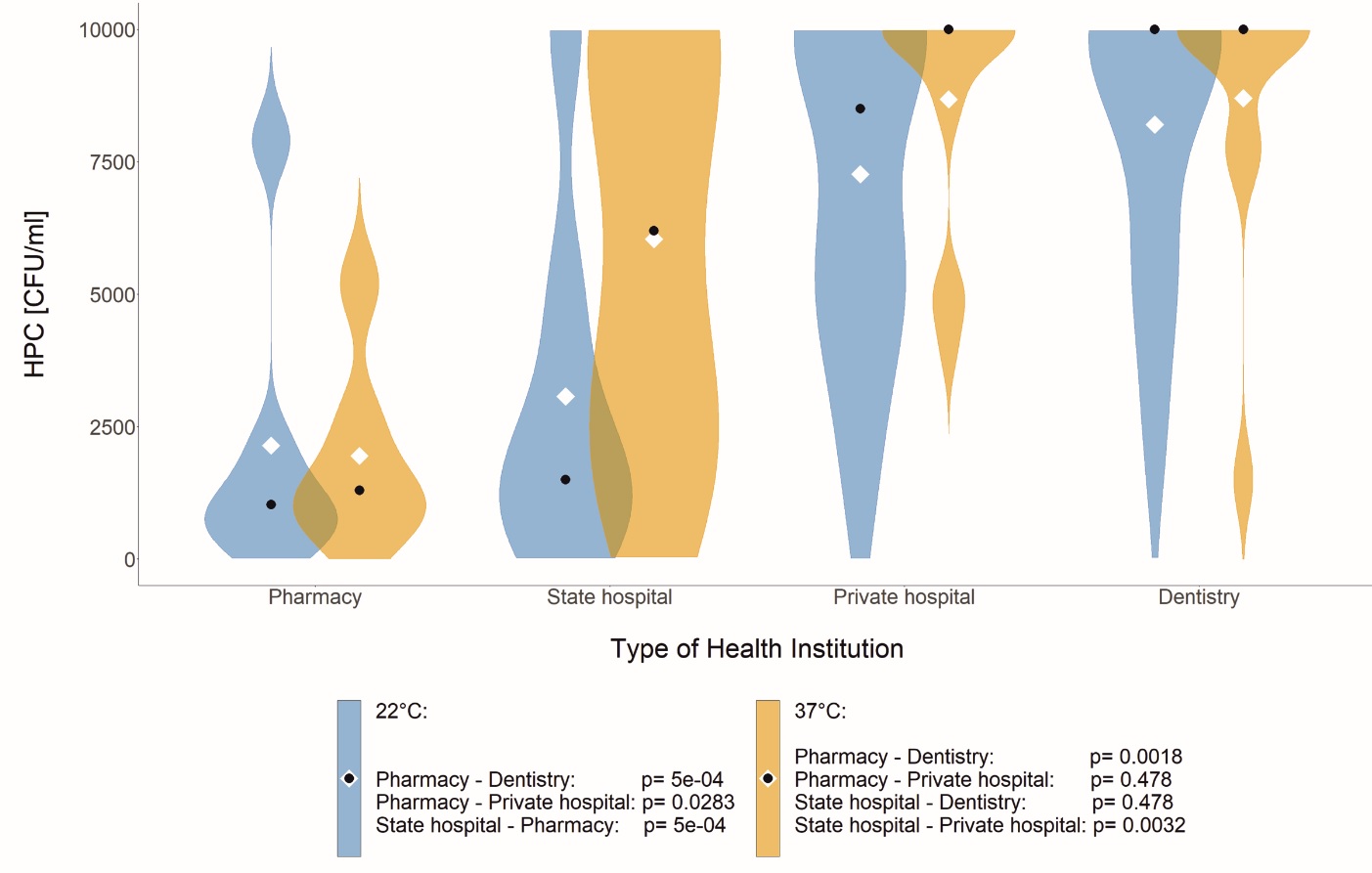

Supplement: Supplementary file 1 [file pathogens-10-00871-s001.zip › Figure S5.jpg]

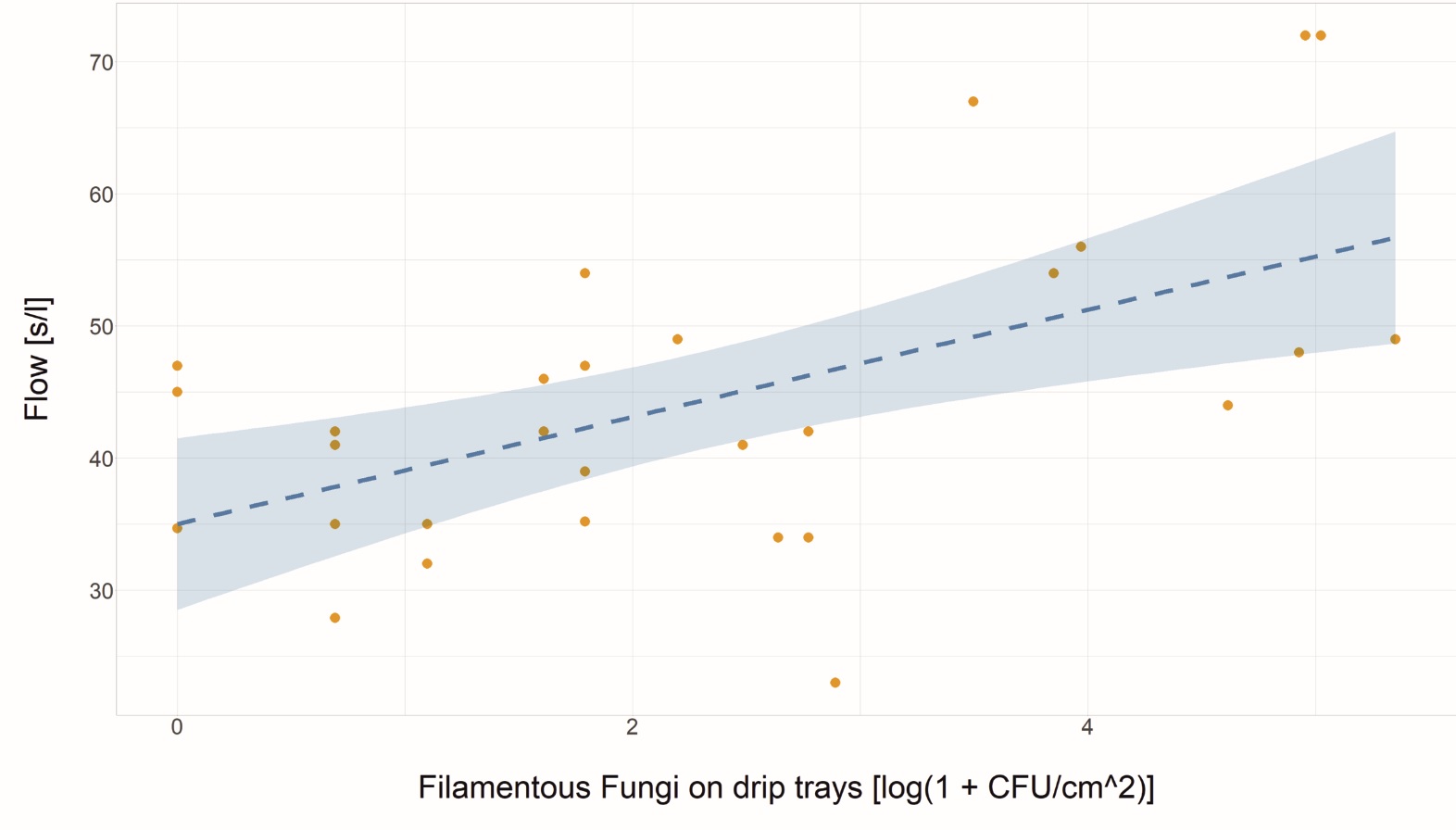

Supplement: Supplementary file 1 [file pathogens-10-00871-s001.zip › Figure S6.jpg]
